# Baseline Testosterone Predicts Body Composition and Metabolic Response to Testosterone Therapy

**DOI:** 10.3389/fendo.2022.915309

**Published:** 2022-07-11

**Authors:** FNU Deepika, Elliot Ballato, Georgia Colleluori, Lina Aguirre, Rui Chen, Clifford Qualls, Dennis T. Villareal, Reina Armamento-Villareal

**Affiliations:** ^1^ Division of Endocrinology Diabetes and Metabolism at Baylor College of Medicine, Houston, TX, United States; ^2^ Department of Medicine, Michael E. DeBakey Veterans Affairs (VA) Medical Center, Houston, TX, United States; ^3^ Division of Endocrinology, University of New Mexico School of Medicine, Albuquerque, NM, United States; ^4^ Department of Medicine, New Mexico Veterans Affairs (VA) Health Care System, Albuquerque, NM, United States; ^5^ Biomedical Research Institute of New Mexico, Albuquerque, NM, United States

**Keywords:** testosterone, body composition, HbA1c, bone turn over markers (BTM), hypogonadism

## Abstract

**Context:**

Male hypogonadism adversely affects body composition, bone mineral density (BMD), and metabolic health. A previous report showed that pre-treatment testosterone (T) levels of <200 ng/dl is associated with greater improvement in spine BMD with T therapy. However, to date, there is no study that investigates whether baseline T levels also influence body composition and metabolic response to T therapy.

**Objective:**

The aim of this study is to determine if there are differences in the changes in body composition, metabolic profile, and bone turnover markers, in addition to BMD, in response to T therapy in men with a baseline T level of <264 ng/dl compared to those with levels ≥264 ng/dl.

**Methods:**

This is a secondary analysis of a single-arm, open-label clinical trial (NCT01378299) on pharmacogenetics of response to T therapy conducted between 2011 and 2016 involving 105 men (40–74 years old), with average morning T < 300 ng/dl, given intramuscular T cypionate 200 mg every 2 weeks for 18 months. Subjects were divided into those with baseline T levels of <264 ng/dl (*N* = 43) and those with ≥264 ng/dl (*N* = 57). T and estradiol (E2) were measured by liquid chromatography/mass spectrometry; serum bone turnover markers (C-telopeptide [CTX], osteocalcin, and sclerostin), adiponectin, and leptin were measured by enzyme-linked immunosorbent assay; glycated hemoglobin (HbA1c) was measured by high-performance liquid chromatography; and areal BMD and body composition was measured by dual-energy x-ray absorptiometry (DXA).

**Results:**

Men with T < 264 ng/dl showed greater increases in total fat-free mass (FFM) at 18 months compared to those with T ≥ 264 ng/dl (4.2 ± 4.1 vs. 2.7 ± 3.8%; *p* = 0.047) and unadjusted appendicular FFM at 6 and 18 months (8.7 ± 11.5 vs. 4.4 ± 4.3%, 7.3 ± 11.6 vs. 2.4 ± 6.8%; *p* = 0.033 and *p* = 0.043, respectively). Men with T ≥ 264 ng/dl showed significant decreases in HbA1c at 12 months (−3.1 ± 9.2 vs. 3.2 ± 13.9%; *p* = 0.005), fasting glucose at 18 months (−4.2 ± 31.9 vs. 13.0 ± 57.3%; *p* = 0.040), LDL at 6 months (−6.4 ± 27.5 vs. 12.8 ± 44.1%; *p* = 0.034), and leptin at 18 months (−40.2 ± 35.1 vs. −27.6 ± 31.0%; *p* = 0.034) compared to those with T < 264 ng/dl. No significant differences in BMD and bone turnover markers were observed.

**Conclusion:**

T therapy results in improvement in body composition irrespective of baseline T levels but T < 264 ng/dl is associated with greater improvement in FFM, whereas a T level of ≥264 ng/dl favors improvement in metabolic profile.

## Introduction

There is an emerging role of testosterone (T) therapy in the management of obesity in men (1). It is well known that overt hypogonadism in men adversely affects body composition, bone density, and metabolic health. Several cross-sectional and longitudinal studies have reported the association between low serum T and risk of metabolic syndrome or Type 2 diabetes (T2D) (2–9). As a result, there is growing interest in studying the role of T therapy in cardiometabolic health. To date, studies looking at the effects of T therapy on body composition have provided equivocal results, with some showing improvement in waist circumference, visceral fat (10–12), and subcutaneous fat (13), and others with no such improvement (14). Indeed, most of these are short term and underpowered, and only a few are longitudinal.

The role of T in the improvement of BMD is well established (15–17). To this point, however, only one study by Synder et al. looked at different pre-treatment serum T concentrations and showed that T replacement therapy showed the greatest improvement in BMD at the lumbar spine in patients with pre-treatment testosterone levels of <200 ng/dl (18). This study was done decades ago when the measurement of serum T was not standardized yet. Recently, T assay has been standardized and LC/MS is considered as the gold standard because of its higher specificity, sensitivity, and precision. In addition, the cutoff for low T has been changed to 264 ng/dl from 300 ng/dl used in the previous guidelines (19). Hence, it remains unknown if those who received T outside of the current guidelines benefited from T therapy. The objective of this secondary analysis is to determine if there are differences in the changes in body composition, metabolic parameters, and bone turnover markers, in addition to BMD, in response to T therapy in men with baseline T level measured by LC/MS and defined as low by the current Endocrine Society guidelines, i.e., T < 264 ng/dl [88] compared to those with levels ≥264 ng/dl. We hypothesize that T therapy in men with pre-treatment testosterone levels of <264 ng/dl will show greater benefit in terms of improvement in body composition, and metabolic and bone parameters.

## Materials and Methods

### Study Design and Study Population

This study is a secondary analysis of longitudinal data obtained from hypogonadal male veterans who volunteered to participate in an open label clinical trial investigating the effect of genetics on T therapy (20), carried out from October 2011 to November 2016 (ClinicalTrials.gov identifier: NCT01378299). Information regarding study design, inclusion and exclusion criteria of the subjects, as well as details of T therapy have been published elsewhere (21). In brief, hypogonadism was defined as an average total T of <300 ng/dl from two samples taken in the morning. The study was conducted at the University of New Mexico VA Health Care System (NMVAHCS) and at the Michael E. DeBakey VA Medical Center (MEDVAMC) in accordance with guidelines of the Declaration of Helsinki for the ethical treatment of human subjects. The protocol was approved by the Institutional Review Boards of the University of New Mexico and of Baylor College of Medicine. Participants were recruited from patients attending the Endocrine, Urology, and Primary Care Clinics of the NMVAHCS and MEDVAMC. Recruitment was accomplished either through flyers or letters to physicians about patients who may qualify for the study. Written informed consent was obtained from each subject. The inclusion criteria were male patients between 40 and 75 years of age with no medical problems that may prevent them from finishing the study. Exclusion criteria included treatment with bone-acting drugs (e.g., bisphosphonates, teriparatide, denosumab, glucocorticoids, sex steroid compounds, selective estrogen receptor modulators, androgen deprivation therapy, and anticonvulsants) and finasteride. Additional exclusion criteria included osteoporosis and history of fragility fractures or diseases known to affect bone metabolism, such as hyperparathyroidism, chronic liver disease, uncontrolled or untreated hyperthyroidism, and significant renal impairment (creatinine of >1.5 mg/dl). Those with a history of prostate cancer, breast cancer, and untreated sleep apnea also met the criteria for exclusion.

### Testosterone Therapy

Therapy consisted of intramuscular injection of 200 mg of T cypionate administered every 2 weeks. The dose was subsequentially adjusted to reach the T serum target level of 17.3 to 27.7 nmol/L (500–800 ng/dl). However, after the third year of the study, upon the direction of the FDA, this target was changed to 17.3 to 20.8 nmol/L (300–600 ng/dl). This change affected the last 6 months of data for 16 subjects at NMVAHCS and of all 15 subjects at MEDVAMC. We did not detect significant difference in T levels among those affected and those not affected by the change as published previously (21). For all subjects, safety monitoring and dose adjustments were performed based on T and hematocrit (Hct) levels; dose adjustment modalities have been published elsewhere (21). The participants were given T therapy for 18 months.

### Biochemical Measurements

Fasting blood samples were collected at baseline; serum samples were extracted and stored at –80°C until analysis except for the baseline screening of T levels. Baseline serum T represents an average from 2 determinations taken 30 min apart between 8:00 and 11:00 a.m., and measured using automated immunoassay, detection range 10 to 3,200 ng/dl (Vitros^®^, Ortho Clinical Diagnostics, Rochester, NY). The coefficient of variation (CV) for T assay was ≤20% for T < 50 ng/dl and ≤ 10% for T 200 to 1,000 ng/dl. At the end of the study, T and E2 for each time point were measured by liquid chromatography/mass spectrometry (Mayo Clinic Laboratories, Mayo Clinic, Rochester, MN, USA). T intra-assay CVs were 7.4%, 6.1%, 9.0%, 2.3%, and 0.9% at 0.65, 4.3, 48, 118, and 832 ng/dl, respectively. Interassay CVs were 8.9%, 6.9%, 4.0%, 3.6%, and 3.5% at 0.69, 4.3, 45, 117, and 841 ng/dl, respectively. The detection range was 0.5 to 2,000 ng/dl. For analyses in this study, T values by LC/MS from blood obtained at screening were used (see *Statistical Methods* section). E2 assay sensitivity was 0.23 pg/ml to 405 pg/ml, intra-assay CV was 1.4% to 11.8%, and interassay CV was 4.8% to 10.8% (20). Luteinizing hormone (LH) and follicle-stimulating hormone (FSH) were assessed by a third-generation chemiluminescent assay, while (22) alkaline phosphatase (ALP) was assessed by Vitros Systems at the Michael E. DeBakey and Albuquerque VA Medical Centers. Fasting glucose was measured using a Unicel DxC 800 auto-analyzer (Beckman Coulter, Fullerton, CA, USA) and HbA1c by high-performance liquid chromatography (Tosoh G8, South San Francisco, CA, USA). The following were measured using enzyme-linked immunosorbent assay kits: C-terminal telopeptide of type I collagen (CTX), marker of bone resorption (Crosslaps; Immunodiagnostic System Inc., Gaithersburg, MD, USA), osteocalcin, marker of bone formation (Metra OC; Quidel Corporation, San Diego, CA, USA), sclerostin (TECO medical Sclerostin HS Enzyme Immunoassay Kit, Quidel Corp, San Diego, CA, USA), and adiponectin (Quantikine; R&D Systems; CV 5.5%). The CVs for the above assays in our laboratory are <10% and <3.5% for HbA1c. RIA kits were used to measure leptin (Leptin HL-81K; Linco Research Inc; CV 5.6%).

### Body Mass Index

Body weight was measured using a standard weighing scale and height was obtained using a stadiometer. BMI was calculated as body weight in kilograms (kg) divided by the square of the height in meters (m^2^) and expressed as kg/m^2^.

### Body Composition

Total body mass, lean body mass (mineral-free and fat free), fat mass, and truncal fat were measured by whole body DXA (Enhanced Whole Body Software version 11.2; Hologic, Inc.) as previously described (23). The percentages of whole and regional fat mass (% fat) were obtained from the estimated readings given by the machine for each region of interest. The percentage of total and regional lean mass (% lean) was calculated as lean mass/total or regional mass. We used percentages of fat and lean body mass to correct for body size in the study population. Fat-free mass was calculated by adding whole body bone mineral content to the lean mass (22). The CV for lean mass and fat mass in our laboratory is 1.5% (22). BMI and body composition were assessed at baseline, 6, 12, and 18 months.

### Areal Bone Mineral Density by Dual Energy X-Ray Absorptiometry

Areal BMD was measured by DXA of the lumbar spine and proximal femur using Hologic Discovery (Hologic Inc, Bedford, MA, USA). Regions of interest in the femur include the total hip and femoral neck. The CVs at our center are ~1.1% for the lumbar spine and 1.2% for the proximal femur (21).

### Statistical Methods

For this secondary study, we first determine the T cutoff score, which divides the sample size into two groups based on body composition at baseline. Screening for the parent clinical trial was based on an automated T immunoassay; however, this secondary analysis is based on the LC/MS assay done at the end of the study from stored baseline samples. Using a nonlinear regression threshold model of cutoff score of baseline T values that maximizes the separation of Y = total FFM and of Y = total fat into two groups, the optimum cutoff score was 200 ng/dl for the immunoassay T and equivalently 262 ng/dl for the mass spectrometry T for both outcomes (**Supplementary Figure 1**). Since it caused no difference in separation of our body composition outcomes, we use the cutoff score of 264 ng/dl as defined by the Endocrine Society for the diagnosis of hypogonadism [88]. Hence, we grouped our participants to those with T < 264 ng/dl and T ≥ 264 ng/dl.

Baseline values, percent changes, and absolute changes from baseline data are presented as mean ± SD in the tables and means ± SE in the figures. To test whether the changes in body composition, metabolic parameters, and BMD/bone turnover markers are influenced by baseline T levels (as shown above), these 3 domains are considered separately (**Supplementary Figure 2**). The analysis for each variable between the two T levels at each visit was done by analysis of covariance (ANCOVA) with the 2 testosterone levels as a grouping factor and with baseline measure of each variable as a covariate.

All results for body composition and BMD were additionally adjusted for age and BMI. The results of metabolic, hormonal, and safety profile, and bone turnover markers were adjusted for baseline only. Unadjusted *p*-values are explicitly indicated. A *p*-value of 0.05 or less is considered statistically significant. Data were managed using Excel 2013 (Microsoft, Redmond, WA) and analyzed using SAS version 9.3 (SAS Institute, Inc., Cary, NC, USA).

## Results

### Baseline Characteristics

A total of 105 subjects participated in the study; 100 subjects had T level by mass spectrometry available. Forty-three had T levels <264 ng/dl and 57 had T levels of ≥264 ng/dl. The baseline characteristics of all the subjects are listed in **Table 1**. Men with T < 264 ng/dl had higher weight (105.1 ± 17.0 kg vs. 97.8 ± 16.5 kg, *p* = 0.033) and BMI (33.8 ± 5.8 kg/m² vs. 31.4 ± 5.0 kg/m², *p* = 0.035) compared to those with T ≥ 264 ng/dl. The remaining characteristics did not differ significantly between the two groups. Out of the 105 subjects in the entire study, 49 had T2D, while 56 did not have diabetes. Among the 49 patients with T2D, 42 were treated with blood glucose-lowering agents: 2 were treated with sulfonylureas alone, 8 with metformin alone, 6 were on sulfonylurea + metformin, 7 were on insulin alone, 1 patient was on a combination of sulfonylurea + insulin, 11 were on metformin + insulin, 4 were on a combination of sulfonylurea + metformin + insulin, 2 were on metformin + incretin agonists, and 1 patient was on combination of metformin + insulin + incretin agonist (20). A total of 70 subjects completed the study (33 in the group with T < 264 ng/dl and 37 in the group with T ≥ 264 ng/dl).

**Table 1:** Demographic and baseline clinical characteristics.

Characteristics	T < 264 ng/dl (*n* = 43)	T ≥ 264 ng/dl (*n* = 57)	*p*-value
**Age (years)**	59.0 (6.8)	59.9 (9.5)	0.603
** BMI**	33.8 (5.8)	31.4 (5.0)	**0.035**
**Weight (kg)**	105.1 (17.0)	97.8 (16.5)	**0.033**
**Vitamin D 25-hydroxy (ng/ml)**	27.0 (9.1)	28.1 (10.4)	0.603
** PTH (pg/ml)**	50.5 (22.4)	48.4 (20.9)	0.651
** ALP (U/L)**	81.4 (22.2)	81.1 (21.0)	0.949
** T2D (%)**	21 (48)	24 (42.1)	0.547
** HbA1c (%)**	6.7 (1.5)	6.6 (1.7)	0.659
**Sedentary lifestyle, *n* (%)**	36 (92.3)	39 (84.8)	0.600
**Smoking status (yes), *n* (%)**	16 (37.2)	22 (38.6)	1.000
** HTN, *n* (%)**	30 (69.8)	35 (61.4)	0.406
** HLD, *n* (%)**	30 (69.8)	38 (66.7)	0.830
**Statin use, *n* (%)**	20 (46.5)	23 (40.35)	0.549
** CVD, *n* (%)**	4 (9.3)	11 (19.3)	0.387
** OSA, *n* (%)**	14 (32.6)	12 (21.1)	0.421

Values are expressed as means (SD), except T2D, sedentary lifestyle, smoking status, HTN, HLD, statin use, CVD, and OSA values expressed as n (%).

Bolded p-values are statistically significant. T, testosterone; PTH, parathyroid hormone; ALP, alkaline phosphatase; T2D, type 2 diabetes; HbA1c, glycated hemoglobin; HTN, hypertension; HLD, hyperlipidemia; CVD, cardiovascular disease; OSA, obstructive sleep apnea.

### Body Composition Outcomes

**Figure 1:**
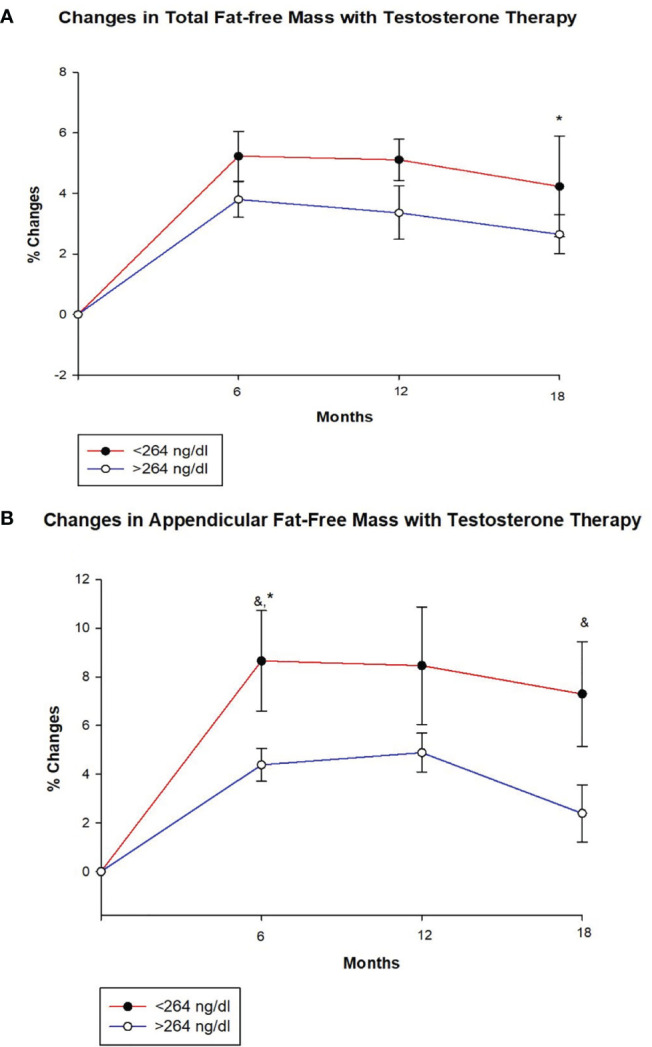
Changes (%) in total and appendicular fat free mass with testosterone (T) therapy according to baseline testosterone level. T < 264 ng/dL (red line), T ≥264 ng/dL (blue line). & Unadjusted P < 0.05, *Adjusted P value < 0.05, adjusted for age and body mass index. **(A)** Total FFM increased in both groups at 6, 12 and 18 months, but the increase at 18 months was greater for those with T < 264 ng/dL compared to those with T ≥264 ng/dL (adjusted p = 0.047). **(B)** Appendicular FFM increased uniformly in both groups at 6, 12 and 18 months. However, the increase was greater in group with T < 264 ng/dL at 6 months (unadjusted p = 0.033 and adjusted p = 0.034) and 18 months (unadjusted p = 0.043) compared to those with T ≥264 ng/dL.

**Table 2** shows changes in body composition over the course of 18 months of T therapy between the two groups. Men with T < 264 ng/dl showed a greater increase in total FFM at 18 months compared to men with T ≥ 264 ng/dl (4.2 ± 4.1% vs. 2.7 ± 3.8%, *p* = 0.047) (**Figure 1A** and **Table 2**). Furthermore, those with T < 264 ng/dl showed a greater increase in unadjusted FFM at the appendicular site at both 6 and 18 months compared to those with T ≥ 264 ng/dl (8.7 ± 11.5% vs. 4.4 ± 4.3%, *p* = 0.033 and 7.3 ± 11.6% vs. 2.4 ± 6.8%, *p* = 0.043, respectively) (**Figure 1B**, **Table 2**). However, after adjusting for age and BMI, only the 6-month comparison remained statistically significant (*p* = 0.034), while the significance at 18 months became borderline (*p* = 0.082). Truncal FFM did not change significantly between the two groups.

**Table 2:** Changes in body composition with testosterone therapy from baseline to 18 months between the two groups.

Body Composition Outcomes	T < 264 ng/dl (*n* = 43)	T ≥ 264 ng/dl (*n* = 57)	*p*-values	
			Unadjusted	Adjusted
**Total FFM (kg)**				
Baseline	68.2 (7.2)	66.2 (8.5)	0.219	
6 months % Δ	5.2 (4.6) 3.5 (3.0)	3.8 (3.7) 2.4 (2.4)	0.142	0.101
12 months % Δ	5.1 (3.7) 3.6 (2.3)	3.4 (4.0) 2.6 (3.5)	0.233	0.266
18 months % Δ	4.2 (4.1) 3.6 (5.8)	2.7 (3.8) 1.8 (2.5)	0.121	**0.047**
**Appendicular FFM (kg)**				
Baseline	30.5 (4.5)	29.9 (4.1)	0.515	
6 months % Δ	8.7 (11.5) 2.5 (2.8)	4.4 (4.3) 1.2 (1.3)	**0.033**	**0.034**
12 months % Δ	8.5 (12.3) 2.3 (2.8)	4.9 (4.5) 1.5 (1.3)	0.138	0.264
18 months % Δ	7.3 (11.6) 2.0 (2.6)	2.4 (6.8) 0.7 (2.1)	**0.043**	0.082
**Truncal FFM (kg)**				
Baseline	33.4 (3.4)	32.4 (4.6)	0.231	
6 months % Δ	4.2 (5.4) 1.3 (1.7)	5.0 (10.3) 1.6 (3.6)	0.702	0.751
12 months % Δ	3.9 (4.8) 1.2 (1.5)	2.8 (5.3) 0.9 (1.6)	0.434	0.243
18 months % Δ	3.3 (4.8) 1.0 (1.6)	3.0 (4.4) 0.9 (1.4)	0.830	0.341
**Total Fat Mass (kg)**				
Baseline	35.3 (11.2)	31.3 (10.3)	0.071	
6 months % Δ	−6.8 (8.40) −2.2 (2.9)	−6.0 (5.1) −1.9 (2.0)	0.599	0.574
12 months % Δ	−7.9 (8.00) −2.6 (2.7)	−7.8 (10.3) −2.2 (3.8)	0.955	0.933
18 months % Δ	−5.0 (10.1) −1.4 (3.4)	−6.2 (9.8) −1.7 (3.3)	0.631	0.839
**Appendicular Fat Mass (kg)**				
Baseline	15.0 (4.5)	13.5 (4.1)	0.099	
6 months % Δ	−7.1 (10.3) −0.9 (1.4)	−6.8 (4.8) −0.9 (0.8)	0.859	0.633
12 months % Δ	−8.4 (7.3) −1.2 (1.1)	−6.5 (9.5) −0.7 (1.4)	0.399	0.200
18 months % Δ	−7.2 (10.1) −0.9 (1.4)	−7.8 (8.7) −0.9 (1.1)	0.790	0.938
**Truncal Fat Mass (kg)**				
Baseline	19.0 (7.0)	16.5 (6.4)	0.074	
6 months % Δ	−7.9 (10.1) −1.4 (1.9)	−5.7 (8.0) −1.0 (1.4)	0.303	0.358
12 months % Δ	−7.5 (11.4) −1.3 (2.1)	−7.5 (11.6) −1.2 (2.3)	0.985	0.994
18 months % Δ	−3.7 (12.3) −0.6 (2.2)	−4.8 (12.9) −0.8 (2.2)	0.729	0.811
**Total Lean Mass (kg)**				
Baseline	65.5 (7.0)	63.6 (8.3)	0.241	
6 months % Δ	5.5 (4.8) 3.5 (3.1)	3.9 (3.9) 2.4 (2.4)	0.136	0.096
12 months % Δ	5.2 (3.9) 3.3 (2.5)	3.6 (4.1) 2.3 (2.6)	0.133	0.104
18 months % Δ	4.3 (4.3) 2.7 (2.7)	2.9 (3.9) 1.8 (2.5)	0.171	0.074
**Appendicular Lean Mass (kg)**				
Baseline	29.2 (4.2)	28.2 (4.0)	0.253	
6 months % Δ	7.0 (5.9) 2.0 (1.8)	5.9 (9.2) 1.5 (2.1)	0.563	0.446
12 months % Δ	6.7 (5.6) 1.9 (1.5)	6.6 (9.9) 1.8 (2.3)	0.987	0.939
18 months % Δ	5.8 (5.1) 1.6 (1.4)	9.3 (29.9) 2.3 (7.0)	0.535	0.500
**Truncal Lean Mass (kg)**				
Baseline	32.7 (3.3)	31.8 (4.5)	0.242	
6 months % Δ	4.3 (5.5) 1.3 (1.7)	3.6 (4.8) 1.1 (1.5)	0.559	0.378
12 months % Δ	3.9 (4.9) 1.2 (1.5)	2.8 (5.4) 0.8 (1.7)	0.391	0.220
18 months % Δ	3.3 (4.9) 1.0 (1.6)	3.0 (4.4) 0.9 (1.4)	0.834	0.348

Values are means (SD); change scores are reported as percent (%) change and absolute change (Δ) from baseline value. Between-group p-values are reported as unadjusted by t-tests and adjusted for baseline, age, and BMI by analysis of covariance (ANCOVA). Bolded p-values are statistically significant. FFM, fat-free mass; T, testosterone.

Both groups of men showed a decline in total as well as regional (truncal and appendicular) fat mass over time. However, there were no significant between-group differences in total and regional (truncal and appendicular) fat mass. An increase in total and regional (truncal and appendicular) lean mass was seen in both groups at all time points but no between-group differences were observed.

The changes in BMI did not differ significantly between the two groups (data not shown).

### Metabolic Profile and Adipokines

#### Hemoglobin A1c and Lipid Profile

**Figure 2:**
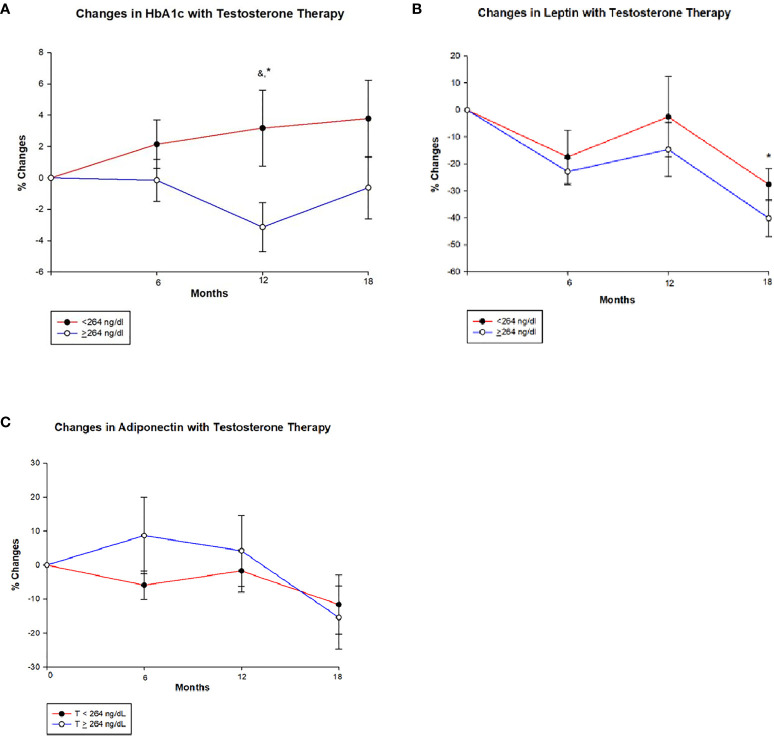
Changes (%) in glycated haemoglobin (HbA1c), leptin and adiponectin with testosterone (T) therapy according to baseline testosterone level. T <264 ng/dL (red line), T ≥264 ng/dL (blue line). & Unadjusted P < 0.05, *Adjusted P value <0.05, adjusted for baseline. **(A)** Among men with T <264 ng/dL, HbA1c shows a non-significant trend towards increase from baseline value at 6, 12 and 18 months, whereas men with T ≥264 ng/dL show a significant reduction in HbA1c at 12 months (Unadjusted p = 0.030 and adjusted p = 0.005) compared to those with < 264 ng/dL. However this significance is lost at 18 months in those T ≥264 ng/dL. **(B)** In both groups leptin levels decline from baseline at 6 months and appear to stabilize at 12 months, but at 18 months there is a greater decline in those with T ≥264 ng/dL compared to <264 ng/dL (p = 0.034). **(C)** Adiponectin levels shows an interesting trend, men in group with T < 264 ng/dL show an initial decline followed by a slight increase and a large decline at 18 months, whereas those with T ≥264 ng/dL show an initial increase in adiponectin followed by a steep decline from 12 to 18 months time point. However, no significant between-groups were seen.

**Table 3** shows that HbA1c declined significantly at 12 months in men with T ≥ 264 ng/dl in comparison to those with T < 264 ng/dl (−3.1 ± 9.2% vs. 3.2 ± 13.9%, *p* = 0.005) (**Figure 2A** and **Table 3**). This reduction in HbA1c was, however, less at the 18-month time point resulting in borderline significant difference between the two groups (*p* = 0.082). At 18 months, there was an increase in fasting blood sugar (FBS) in the group with T < 264 ng/dl in contrast to a decrease in those with T ≥ 264 ng/dl resulting in significant between-group differences (13.0 ± 57.3% vs. –4.2 ± 31.9%, *p* = 0.040).

**Table 3:** Changes in metabolic profile and adipokines with testosterone therapy from baseline to 18 months between the two groups.

Outcome Variables	T < 264 ng/dl (*n* = 43)	T ≥ 264 ng/dl(*n* = 57)	*p*-value	
			Unadjusted	Adjusted
**HbA1c (%)**				
Baseline	6.7 (1.5)	6.6 (1.7)	0.664	
6 months % Δ	2.2 (9.0) 0.1 (0.7)	−0.2 (8.8) −0.1 (0.8)	0.264	0.180
12 months % Δ	3.2 (13.9) 0.2 (1.1)	−3.1 (9.2) −0.3 (0.8)	**0.030**	**0.005**
18 months % Δ	3.8 (12.5) 0.2 (0.9)	−0.6 (10.7) −0.1 (1.0)	0.167	0.082
**FBS (mg/dl)**				
Baseline	124.2 (45.7)	120.4 (41.4)	0.663	
6 months % Δ	2.8 (41.4) −0.9 (71.0)	−0.2 (6.2) −0.8 (47.1)	0.691	0.611
12 months % Δ	4.2 (37.9) −0.6 (55.0)	2.2 (31.0) −0.4 (44.2)	0.815	0.489
18 months % Δ	13.0 (57.3) 6.1 (56.2)	−4.2 (31.9) −6.7 (42.0)	0.131	**0.040**
**Total Cholesterol (mg/dl)**				
Baseline	170.5 (44.4)	175.6 (45.0)	0.579	
6 months % Δ	−1.5 (20.2) −6.6 (33.9)	−5.6 (22.0) −13.8 (34.7)	0.387	0.488
12 months % Δ	1.0 (21.6) −3.0 (35.0)	−2.5 (26.9) −9.9 (40.7)	0.567	0.934
18 months % Δ	0.9 (21.1) −2.4 (36.8)	−3.1 (28.8) −13.2 (43.6)	0.555	0.894
**TG (mg/dl)**				
Baseline	188.5 (149.1)	166.6 (83.0)	0.355	
6 months % Δ	1.9 (49.5) −13.1 (112.7)	−1.3 (2.4) −8.8 (70.0)	0.730	0.562
12 months % Δ	22.9 (63.2) 27.4 (96.7)	18.8 (61.4) 15.4 (83.4)	0.786	0.840
18 months % Δ	20.4 (63.7) 21.4 (108.6)	1.2 (47.6) −0.4 (88.7)	0.204	0.208
**LDL (mg/dl)**				
Baseline	95.7 (39.7)	102.0 (36.9)	0.427	
6 months % Δ	12.8 (44.1) 1.9 (30.2)	−6.4 (27.5) −9.4 (27.2)	**0.019**	**0.034**
12 months % Δ	7.1 (48.6) −3.5 (29.1)	−4.4 (27.2) −7.8 (29.0)	0.230	0.339
18 months % Δ	4.6 (45.1) −3.7 (33.5)	−0.2 (32.5) −6.1 (30.0)	0.651	0.850
**HDL (mg/dl)**				
Baseline	40.4 (10.4)	42.6 (14.8)	0.401	
6 months % Δ	−6.8 (13.0) −3.2 (6.1)	−6.6 (19.2) −3.2 (9.7)	0.947	0.792
12 months % Δ	−7.2 (19.7) −3.1 (9.8)	−10.8 (14.6) −5.4 (7.4)	0.383	0.475
18 months % Δ	−8.1 (17.1) −3.3 (7.1)	−15.3 (11.1) −7.3 (7.0)	0.063	0.097
**Leptin (ng/ml)**				
Baseline	3.1 (2.3)	2.4 (1.6)	0.125	
6 months % Δ	−17.4 (56.2) −0.8 (1.1)	−22.9 (29.4) −0.6 (1.0)	0.612	0.552
12 months % Δ	−2.5 (76.5) −1.2 (2.5)	−14.7 (55.6) −1.0 (1.6)	0.492	0.160
18 months % Δ	−27.6 (31.0) −1.3 (2.0)	−40.2 (35.1) −1.3 (1.3)	0.170	**0.034**
**Adiponectin (µg/ml)**				
Baseline	49.2 (26.6)	48.8 (27.6)	0.945	
6 months % Δ	−5.9 (23.2) −4.6 (13.6)	8.7 (66.8) 1.0 (22.1)	0.254	0.347
12 months % Δ	−1.7 (32.1) −5.3 (16.5)	4.2 (57.1) −0.6 (26.0)	0.644	0.880
18 months % Δ	−11.6 (45.2) −8.3 (20.0)	−15.4 (45.4) −8.3 (17.6)	0.767	0.618

Values are means (SD); change scores are reported as percent (%) change and absolute change (Δ) from baseline value. Between-group p-values are reported as unadjusted by t-tests and adjusted for baseline by analysis of covariance (ANCOVA). Bolded p-values are statistically significant. T, testosterone; BMI, body mass index; FBS, fasting blood sugar; HbA1c, glycated hemoglobin; TG, triglyceride; LDL, low-density lipoprotein; HDL, high-density lipoprotein.

At 6 months, LDL levels declined in men with T ≥ 264 ng/dl compared to the increase in those with T < 264 ng/dl (–6.4 ± 27.5% vs. 12.8 ± 44.1%, *p* = 0.034). Total cholesterol and HDL levels decreased at all time points in both groups with no significant between-group differences. Men with T < 264 ng/dl had an increase in triglycerides (TG) levels by the end of the study, while levels were unchanged among those with T ≥ 264 ng/dl with no significant between-group differences.

#### Adipokines

Leptin levels decreased from baseline at 6, 12, and 18 months in both groups; however, the decrease was significantly greater in men with T ≥ 264 ng/dl compared to those with T < 264 ng/dl at 18 months (−40.2 ± 35.1% vs. –27.6 ± 31.0%, *p* = 0.034) (**Figure 2B** and **Table 3**). Meanwhile, adiponectin decreased at the end of the study in both groups with no significant between-group difference (**Figure 2C** and **Table 3**).

### Bone Outcomes

#### a) Areal Bone Mineral Density (BMD) by DXA

At baseline (**Table 4**), subjects with T < 264 ng/dl had a significantly higher BMD at the lumbar spine and total hip when compared to those with T ≥ 264 ng/dl (1.161 ± 0.15 g/cm^2^ vs. 1.081 ± 0.14 g/cm^2^, *p* = 0.010 and 1.124 ± 0.146 g/cm^2^ vs. 1.039 ± 0.12 g/cm^2^, *p* = 0.002, respectively). Both groups showed a progressive increase in BMD at the lumbar spine, with no significant between-group differences in either unadjusted or adjusted BMD changes. Similarly, there were no significant between-group differences in BMD changes (unadjusted and adjusted) at the total hip and femoral neck.

**Table 4:** Changes in bone mineral density with testosterone therapy from baseline to 18 months between the two groups.

Outcome Variables	T < 264 ng/dl (*n* = 43)	T ≥ 264 ng/dl (*n* = 57)	*p*-value	
			Unadjusted	Adjusted
**Lumbar spine (g/cm^2^)**				
Baseline	1.161 (0.15)	1.081 (0.14)	**0.010**	
6 months % Δ	2.4 (3.3) 0.029 (0.04)	1.6 (2.8) 0.017 (0.03)	0.276	0.393
12 months % Δ	3.6 (3.0) 0.043 (0.04)	2.2 (2.5) 0.023 (0.03)	0.056	0.101
18 months % Δ	4.6 (3.4) 0.055 (0.04)	3.2 (3.5) 0.033 (0.03)	0.107	0.167
**Total hip (g/cm^2^)**				
Baseline	1.124 (0.146)	1.039 (0.12)	**0.002**	
6 months % Δ	−0.1 (3.0) −0.002 (0.03)	0.8 (2.9) 0.009 (0.03)	0.193	0.275
12 months % Δ	0.3 (3.7) 0.001 (0.04)	0.5 (3.5) 0.004 (0.04)	0.779	0.674
18 months % Δ	0.9 (3.3) 0.007 (0.04)	0.9 (2.9) 0.009 (0.03)	0.938	0.576
**Femoral neck (g/cm^2^)**				
Baseline	0.838 (0.11)	0.806 (0.12)	0.194	
6 months % Δ	−0.4 (5.0) −0.003 (0.04)	0.1 (4.0) 0.001 (0.03)	0.610	0.434
12 months % Δ	−0.5 (3.3) −0.004 (0.03)	0.7 (4.7) 0.006 (0.04)	0.230	0.167
18 months % Δ	−0.2 (4.3) −0.003 (0.03)	0.6 (5.4) 0.003 (0.04)	0.501	0.531

Values are means (SD); change scores are reported as both percent (%) change and absolute change (**Δ**) from baseline value, and baseline values are the observed means (SD). Between-group p-values are reported as unadjusted by t-tests and adjusted for baseline, age, and BMI by analysis of covariance (ANCOVA). Bolded p-values are statistically significant. T, testosterone.

#### b) Bone Turnover Markers (BTM)

**Table 5** shows that there were no significant between-group differences in changes in osteocalcin, CTX, and sclerostin levels.

**Table 5:** Changes in bone turnover markers with testosterone therapy from baseline to 18 months between the two groups.

Outcome Variables	T < 264 ng/dl (*n* = 43)	T ≥ 264 ng/dl (*n* = 57)	*p*-value	
			Unadjusted	Adjusted
**Osteocalcin (ng/ml)**				
Baseline	5.8 (3.9)	7.0 (4.8)	0.220	
6 months % Δ	3.5 (55.1) −1.2 (3.4)	−4.7 (59.8) −0.9 (4.0)	0.535	0.939
12 months % Δ	31.1 (118.6) −0.6 (4.0)	−7.4 (63.3) −1.9 (4.2)	0.109	0.397
18 months % Δ	25.8 (107.2) −0.9 (3.5)	−9.3 (46.4) −1.8 (4.8)	0.099	0.273
**CTX (ng/ml)**				
Baseline	0.3 (0.2)	0.3 (0.2)	0.839	
6 months % Δ	−22.9 (42.0) −0.1 (0.1)	−19.9 (58.5) −0.1 (0.1)	0.812	0.604
12 months % Δ	−4.1 (55.0) −0.0 (0.1)	−4.9 (43.0) −0.1 (0.1)	0.486	0.842
18 months % Δ	−3.9 (60.2) −0.1 (0.2)	16.6 (71.8) 0.0 (0.2)	0.463	0.209
**Sclerostin (ng/ml)**				
Baseline	0.8 (0.2)	0.8 (0.2)	0.921	
6 months % Δ	0.5 (33.8) −0.0 (0.3)	−0.3 (35.5) −0.0 (0.3)	0.931	0.751
12 months % Δ	−1.2 (36.8) −0.1 (0.3)	−3.2 (41.6) −0.1 (0.4)	0.861	0.985
18 months % Δ	−16.1 (26.9) −0.2 (0.3)	−6.4 (41.4) −0.1 (0.3)	0.345	0.287

Values are means (SD); change scores are reported as both percent (%) change and absolute change (**Δ**) from baseline value. Between-group p-values are reported as unadjusted by t-tests and adjusted for baseline by analysis of covariance (ANCOVA). T, testosterone; CTX, C-terminal telopeptide of type I collagen.

### Hormonal and Safety Profile

By virtue of study design and to keep T levels at a particular goal, men with low baseline T levels of <264 ng/dl had a greater increase in T levels by the end of the study period (18 months) compared to those with T ≥ 264 ng/dl (185.9 ± 231.6 ng/dl vs. 93.2 ± 90.2 ng/dl, *p* = 0.023) (see **Table S1**). Similarly, men with T < 264 ng/dl showed a greater increase in E2 levels at 12 months compared to those with T ≥ 264 ng/dl (154.9 ± 121.9 pg/ml vs. 63.7 ± 100.6 pg/ml, *p* = 0.013).

Hct increase was significantly higher at 6 months in men with T < 264 ng/dl compared to those with T ≥ 264 ng/dl (5.6 ± 3.4% vs. 3.5 ± 2.7%, *p* = 0.001) (see **Table S1**). There were no significant between-group differences in the PSA levels. For subjects with T < 264 ng/dl: 7 experienced a significant increase in Hct defined as ≥55%, 3 had cardiovascular events, and 2 had cerebrovascular events. For those with T ≥ 264 ng/dl: 2 had a significant increase in Hct, 4 had cardiovascular events, and 1 had a prostate enlargement but benign by biopsy. A complete list of adverse events can be found elsewhere (20).

## Discussion

Our study shows that T therapy results in improvement in the different parameters of body composition in men irrespective of their baseline T levels, though men with T < 264 ng/dl, had a significantly greater increase in total and appendicular FFM compared to those with T ≥ 264 ng/dl. However, and in contradiction to our hypothesis, men with T ≥ 264 ng/dl experienced an improvement in several metabolic parameters compared to those with T < 264 ng/dl. On the other hand, no differences in the changes in BMD and bone turnover marker were observed between those with a baseline T level of <264 ng/dl, and those with ≥264 ng/dl.

It is known that sex steroids play a crucial role in energy metabolism, fat redistribution, body composition, and appetite (24, 25). Some of the oldest studies in humans have shown that there is an inverse relationship between T and visceral adipose tissue and that increasing obesity, particularly abdominal obesity, is associated with decreasing T levels in young men (26, 27). Conversely, there is evidence that T levels are positively correlated with insulin sensitivity, and men with low T have a threefold higher prevalence of metabolic syndrome than their eugonadal counterparts (28). A randomized controlled trial (RCT) showed that T treatment in hypogonadal men with T2D increases insulin sensitivity, increases lean mass, and decreases subcutaneous fat (29). There are several other studies showing similar results (30–36); however, to date, there are no studies that evaluate whether the response to T therapy varies according to baseline T levels. In addition, the recent shift in the diagnosis of low T from <300 ng/dl to <264 ng/dl using a more sensitive assay would suggest that some of the patients treated for hypogonadism in the past are not hypogonadal by the current definition. In this study, we compared the changes in body composition in response to T therapy between men with a T of <264 ng/dl and those with a T of ≥264 ng/dl. We found that both groups experienced loss of total and regional fat mass and an increase in total and regional FFM as well as lean mass. However, men with T < 264 ng/dl derived a greater benefit in terms of improvement in total and appendicular FFM compared to those with T ≥ 264 ng/dl.

Interestingly, men with T ≥ 264 ng/dl experienced some improvement in several metabolic parameters such as HbA1c, FBS, LDL, and leptin compared to those with T < 264 ng/dl. In our study, HbA1c and FBS did not change significantly for men with T < 264 ng/dl, but men with T ≥ 264 ng/dl showed significant decline in both parameters with T therapy. Since men with T < 264 ng/dl had relatively lower baseline T levels, it is possible that this group needed more time on adequate T levels to see significant improvement in glycemic control. That T therapy is associated with improvement in glucometabolic profile has been reported by prior studies. Data from an elegant study by Jones et al. showed that T replacement results in improvement in insulin resistance and glycemic control in all men including those with T2D (14). A few other RCTs (11, 37, 38) and retrospective studies (39–41) have shown similar results. Moreover, the data from testosterone trials showed that benefits of T therapy on insulin resistance and fasting glucose extend to men in the elderly age group (42).

In regard to the lipid profile, whether T therapy is beneficial or detrimental revealed inconsistent findings. While total cholesterol decreased, HDL also decreased in both groups with no significant between-group differences. On the other hand, LDL significantly differed between the two groups at 6 months with an increase in LDL in men with T < 264 ng/dl compared to a decline in those with T ≥ 264 ng/dl. Data from two large metanalyses and from a previous observational study shows that T administration to hypogonadal men is associated with a small and insignificant decrease in HDL cholesterol and concomitant declines in total cholesterol, TG, and LDL (43–45). Two small RCTs showed no alteration in HDL with T replacement in hypogonadal men (46, 47). However, unlike our study, these studies did not examine whether baseline T level is a determinant of lipid response to T therapy. In our study, there was no significant difference in baseline statin use between the two groups.

Leptin levels decreased with T therapy in both groups, with a greater reduction in those with T ≥ 264 ng/dl compared to those with T < 264 ng/dl. The decrease in leptin in response to T therapy is not surprising as studies have shown that T administration decreases leptin, both directly by suppressing leptin messenger RNA and leptin secretion from human adipose tissue *in vitro* and indirectly by a reduction in fat mass (48, 49). Indeed, one of the oldest studies showed normalization of leptin levels with T therapy, highlighting that the interaction of T and leptin might be part of a hypothalamic–pituitary–gonadal–adipose tissue axis that is involved in body weight maintenance and reproductive function (50). Nevertheless, it is unclear why men with higher baseline T had greater reduction in leptin in our study.

Adiponectin levels declined progressively in men with T < 264 ng/dl and only by 18 months in those with T ≥ 264 ng/dl. Animal studies found that T therapy reduced plasma adiponectin concentration in both sham-operated and castrated mice (51), and this is either from increased lipolysis or by an increased in beta-adrenergic stimulation (52, 53). Studies in humans also showed that T treatment in hypogonadal men decreased adiponectin levels (13, 51, 54, 55). Regardless, to date, the regulatory role of T on adiponectin remains poorly understood.

Our data also provide further evidence that T treatment improves body composition to a comparable degree between those with T of < or ≥264 ng/dl except for FFM where improvement is significantly better in the low T group. It is important to note that the cutoff of T < 264 ng/dl for the diagnosis of hypogonadism corresponds to <2.5th percentile value, based on harmonization of the normal reference range in a healthy non-obese population of European and American men, 19 to 39 years of age (56). The current guidelines recommend T therapy for improvement in lean mass only in men with HIV and against the use for improvement in glycemic control in men with type 2 diabetes and low T (57). Nevertheless, in clinical practice, we encounter a considerable number of middle age and elderly men with symptoms of hypogonadism who also have metabolic syndrome but with T > 264 ng/dl and in whom T therapy is withheld yet could potentially benefit from T based on the results from our study.

Both groups showed a progressive improvement in BMD at lumbar spine, while BMD at the femoral neck and total hip remained stable with no significant between-group differences. Synder et al. also showed that spine BMD improved with T therapy, although more in men with pre-treatment testosterone levels of <200 ng/dl, with no significant change in BMD at the total hip and femoral neck (18). It is well known that bone changes at the total hip are slow to respond to gonadal steroid treatment compared to the response at the spine (34) and could likely explain the lack of significant improvement at the total hip and femoral neck by 18 months in our study.

Most studies have shown that T therapy decreases markers of bone resorption (58–60). Although our study found no significant differences in changes in BTM between the two groups, a trend towards an increase in osteocalcin and decrease in CTX levels was observed for men with T < 264 ng/dl, while a trend for progressive reduction in osteocalcin and an increase in CTX after an initial decline was observed in those with T ≥ 264 ng/dl. Sclerostin levels, however, appeared to decline in both groups. This decline in sclerostin could have a beneficial effect on bone remodeling as sclerostin inhibits osteoblast differentiation (61). In a study by Khosla’s group, the authors showed that reduction in sclerostin is influenced by estrogen rather than T (62). In our study, the increase in E2 was greater in men with T < 264 ng/dl and may have accounted for the greater reduction in sclerostin in this group. Lastly, men with T < 264 ng/dl had a greater increase in Hct, which is likely from the greater magnitude of T increase. Both groups experienced a non-significant increase in PSA with no difference between the two groups.

Our study is the first one to evaluate if the effects of T therapy on body composition, metabolic profile, and bone are influenced by the baseline T level. So far, only one study by Synder et al. has shown that BMD at the lumbar spine improved the most in men with pre-treatment testosterone levels of <200 ng/dl; however, T was measured before standardization in T assay was implemented. Our study is prospective in nature with a longer follow-up period in comparison to previously published studies (11, 12, 14, 29, 35, 58, 59, 63). There are a few limitations to our study. Firstly, it is a secondary analysis of outcome data from our prior clinical trial. Secondly, for this study, we had limited sample size and a dropout rate of 28% that may have contributed to the lack of between-group differences in some outcomes.

In conclusion, our study shows that men, regardless of baseline T level, derived some benefit from T therapy. Men with T < 264 ng/dl benefit from a greater increase in FFM, while men with T ≥ 264 ng/dl, contrary to our hypothesis, appear to experience greater benefit from the metabolic standpoint, i.e., reduction in HbA1c, glucose, LDL, and leptin. Although prior studies have suggested improvement in insulin sensitivity in men with low T (14, 29), a recommendation to give T to improve the metabolic profile remains controversial (63–65). Our findings support the partial metabolic benefit from T among men with T ≥ 264 ng/dl who, by current guidelines, will not be treated with T. Therefore, the information presented in the manuscript could be valuable for both clinicians and patients in shared decision-making.

## Data Availability Statement

The raw data supporting the conclusions of this article will be made available by the authors, without undue reservation.

## Ethics Statement

The studies involving human participants were reviewed and approved by Institutional Review Boards of Baylor College of Medicine and University of New Mexico. The patients/participants provided their written informed consent to participate in this study.

## Author Contributions

FD and RA-V: conceptualization. FD, CQ, and RA-V: formal analysis. FD, EB, GC, LA, RC, DV, and RA-V: investigation. FD, EB, CQ, DV, and RA-V: writing. FD, EB, GC, LA, RC, DV, and RA-V: reviewing and editing. All authors contributed to the article and approved the submitted version.

## Funding

This study was supported by the VA Merit Review 101CX000424, 101CX001665, and NIH R01 HD093047.

## Author Disclaimer

The contents do not represent the views of the U.S. Department of Veterans Affairs or the United States Government.

## Conflict of Interest

The authors declare that the research was conducted in the absence of any commercial or financial relationships that could be construed as a potential conflict of interest.

## Publisher’s Note

All claims expressed in this article are solely those of the authors and do not necessarily represent those of their affiliated organizations, or those of the publisher, the editors and the reviewers. Any product that may be evaluated in this article, or claim that may be made by its manufacturer, is not guaranteed or endorsed by the publisher.
