# Supplementary figures and images for: Baseline Testosterone Predicts Body Composition and Metabolic Response to Testosterone Therapy
Source: Front Endocrinol (Lausanne). 2022 Jul 11;13:915309. doi: 10.3389/fendo.2022.915309 (PMC9309506; doi:10.3389/fendo.2022.915309)

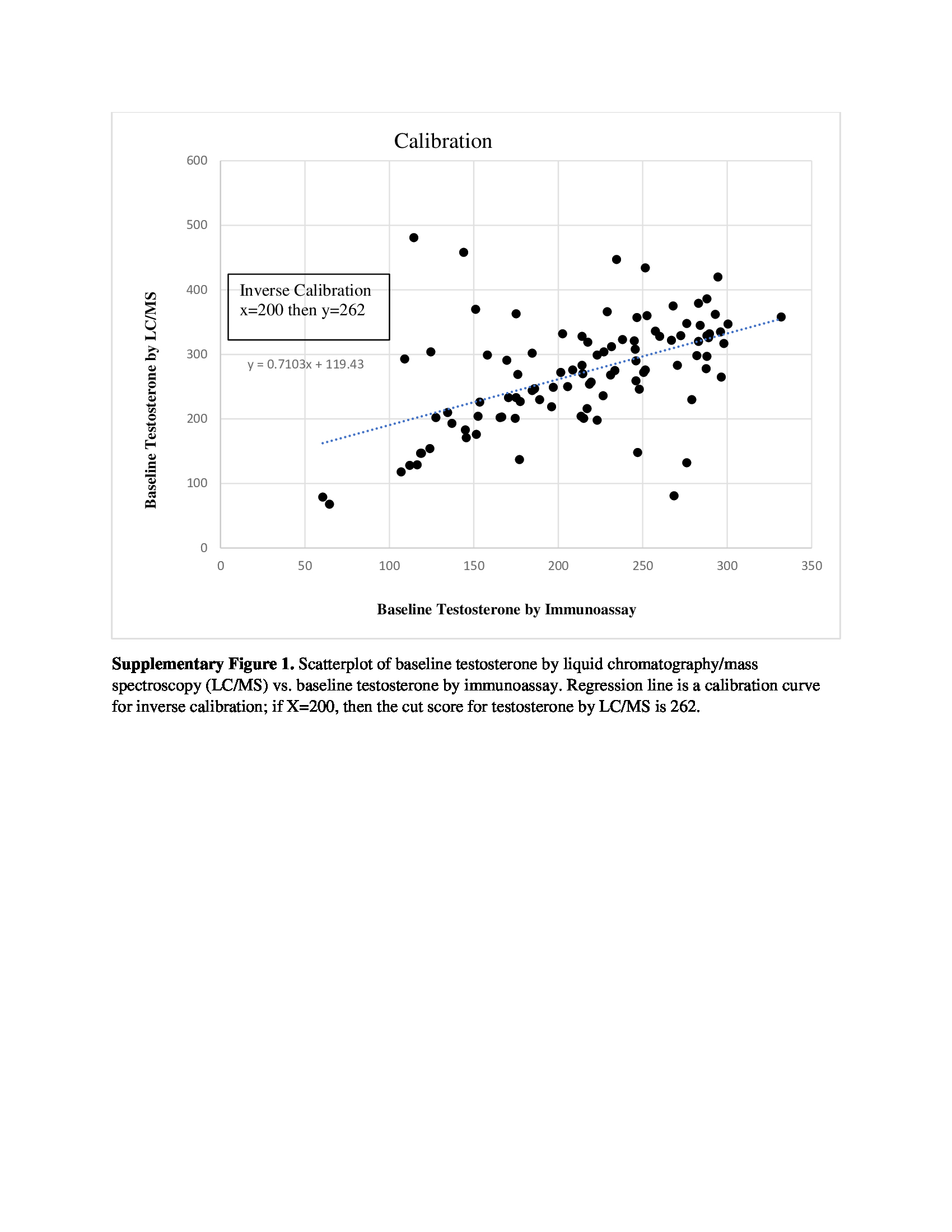

Supplement: Supplementary file 1 [file Image_1.jpeg]

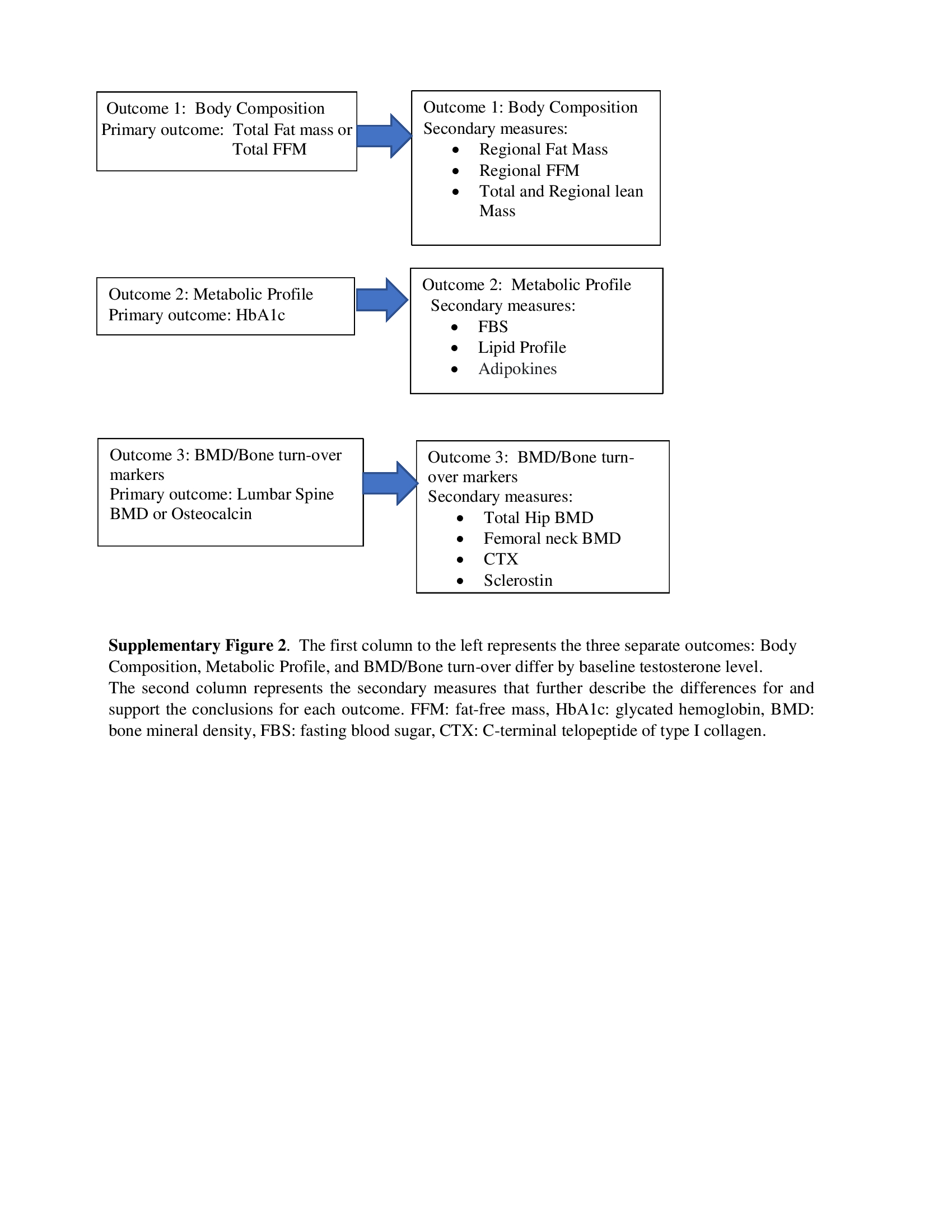

Supplement: Supplementary file 2 [file Image_2.jpeg]
