# Supplementary material for: Baseline Testosterone Predicts Body Composition and Metabolic Response to Testosterone Therapy
Source: Front Endocrinol (Lausanne). 2022 Jul 11;13:915309. doi: 10.3389/fendo.2022.915309 (PMC9309506; doi:10.3389/fendo.2022.915309)
Supplement: Supplementary file 3 [file Table_1.docx]

**Supplementary Table**

**Table S1. Changes in Hormonal and Safety Profile with Testosterone Therapy from Baseline to 18 Months Between the Two Groups**

| Outcome  Variables | T< 264 ng/dL  (n=43) | T ≥ 264 ng/dL  (n=57) | p-value  Unadjusted Adjusted | |
| --- | --- | --- | --- | --- |
| Testosterone |  |  |  |  |
| Baseline (ng/dL) | 193.4 (51.8) | 328.7 (49.6) | **<0.0001** |  |
| Change at 6 mo | 251.9 (227.4) | 113.5 (108.5) | **0.001** | 0.150 |
| Change at 12 mo | 237.1 (235.8) | 94.6 (100.4) | **0.002** | 0.581 |
| Change at 18 mo | 185.9 (231.6) | 93.2 (90.2) | **0.036** | **0.023** |
| Estradiol |  |  |  |  |
| Baseline (pg/mL) | 14.3 (4.0) | 19.1 (6.6) | **0.001** |  |
| Change at 6 mo | 207.0 (155.6) | 120.3 (143.4) | **0.012** | 0.094 |
| Change at 12 mo | 154.9 (121.9) | 63.7 (100.6) | **0.001** | **0.013** |
| Change at 18 mo | 115.3 (119.7) | 89.0 (109.5) | 0.356 | 0.947 |
| Hct (%) |  |  |  |  |
| Baseline | 43.8 (2.9) | 43.6 (2.9) | 0.768 |  |
| Change at 6 mo | 5.6 (3.4) | 3.5 (2.7) | **0.002** | **0.001** |
| Change at 12 mo | 4.3 (4.3) | 3.4 (3.2) | 0.311 | 0.161 |
| Change at 18 mo | 4.2 (3.9) | 4.4 (4.0) | 0.843 | 0.913 |
| PSA |  |  |  |  |
| Baseline (ng/mL) | 1.0 (0.7) | 1.0 (0.7) | 0.664 |  |
| Change at 6 mo | 0.4 (0.5) | 0.4 (0.5) | 0.976 | 1.000 |
| Change at 12 mo | 0.5 (0.5) | 0.3 (0.4) | 0.248 | 0.230 |
| Change at 18 mo | 0.5 (0.7) | 0.5 (0.7) | 0.987 | 0.931 |

Values are means (SD), change scores reported as absolute change from baseline value. Between-group p-values are reported as unadjusted by t-tests and adjusted for baseline by analysis of covariance (ANCOVA). Bolded p-values are statistically significant.

Abbreviations: T: Testosterone, Hct: hematocrit; PSA: prostate-specific antigen.
